# Fear-Conditioning Mechanisms Associated with Trait Vulnerability to Anxiety in Humans

**DOI:** 10.1016/j.neuron.2010.12.034

**Published:** 2011-02-10

**Authors:** Iole Indovina, Trevor W. Robbins, Anwar O. Núñez-Elizalde, Barnaby D. Dunn, Sonia J. Bishop

**Affiliations:** 1 Behavioural and Clinical Neuroscience Institute, Department of Experimental Psychology, University of Cambridge, Downing Street, Cambridge, CB2 3EB, UK; 2 Laboratory of Neuromotor Physiology, Santa Lucia Foundation, via Ardeatina 306, 00179 Rome, Italy; 3 Department of Psychology, Tolman Hall #1650, University of California, Berkeley, CA 94720-1650, USA; 4 Medical Research Council Cognition and Brain Sciences Unit, 15 Chaucer Road, Cambridge, CB2 7EF, UK

## Abstract

Investigations of fear conditioning in rodents and humans have illuminated the neural mechanisms underlying cued and contextual fear. A critical question is how personality dimensions such as trait anxiety act through these mechanisms to confer vulnerability to anxiety disorders, and whether humans' ability to overcome acquired fears depends on regulatory skills not characterized in animal models. In a neuroimaging study of fear conditioning in humans, we found evidence for two independent dimensions of neurocognitive function associated with trait vulnerability to anxiety. The first entailed increased amygdala responsivity to phasic fear cues. The second involved impoverished ventral prefrontal cortical (vPFC) recruitment to downregulate both cued and contextual fear prior to omission (extinction) of the aversive unconditioned stimulus. These two dimensions may contribute to symptomatology differences across anxiety disorders; the amygdala mechanism affecting the development of phobic fear and the frontal mechanism influencing the maintenance of both specific fears and generalized anxiety.

## Highlights

► Amygdala and frontal dysfunction independently linked to vulnerability to anxiety ► Amygdala dysregulation contributes to elevated cued fear in trait anxious subjects ► Anxiety resilience linked to greater vPFC activity during cued and contextual fear ► vPFC role not limited to extinction, but also pre-extinction fear downregulation

## Introduction

Both fear and anxiety are biologically adaptive responses to environmental threat. However, when experienced over a long period of time they can have a devastating effect, as sufferers of anxiety disorders know only too well. So why is it that some of us can overcome the discrete fears and nonspecific anxiety that we experience in our lives more easily than others? Researchers conducting experimental fear conditioning in both humans and animals have argued that dysregulation of the mechanisms underlying the development and maintenance of “conditioned” fear responses may provide an explanation. Basic neuroscience and functional neuroimaging studies have greatly advanced our understanding of these mechanisms (Maren and Quirk, 2004; Sotres-Bayon et al., 2004; Phelps and LeDoux, 2005; Bishop, 2007). Findings from these studies implicate the amygdala in the acquisition and expression of conditioned fear, this being modulated by input from the ventromedial prefrontal cortex (vmPFC) which is thought to inhibit the expression of conditioned fear following extinction training (i.e., when the conditioned stimulus [CS] or acquisition context is repeatedly presented alone, without the aversive unconditioned stimulus [UCS]) (Phelps et al., 2004; Quirk et al., 2006). The hippocampus is also implicated in the contextual modulation of conditioned fear, in particular its extinction (Ji and Maren, 2007). However, it remains to be established how personality characteristics such as high trait anxiety act through these mechanisms to confer a diathesis, or increased vulnerability, for anxiety disorders (Mineka and Oehlberg, 2008). Or to pose the question another way: what differences in neurocognitive function protect low trait anxious individuals from experiencing chronic fear and anxiety? A possibility considered here is that the mechanisms which determine whether certain individuals are more or less anxiety-prone than others might include regulatory processes that fall outside the standard conceptual framework of rodent models of fear conditioning.

Studies investigating differences in neural or cognitive function associated with vulnerability to anxiety have typically sought to distinguish high from low trait anxious individuals in a unitary manner. However, symptom variability across anxiety disorders suggests that there may be at least two dimensions of function associated with risk for these disorders (Mineka and Oehlberg, 2008). In particular, certain anxiety disorders, such as specific phobia, are primarily characterized by cue-specific or phasic fear, while others, e.g., generalized anxiety disorder, are also characterized by diffuse or non-cue-specific anxiety. Experimental fear-conditioning studies suggest that the development of phasic fear and non-cue-specific anxiety may be modeled by cued and contextual fear conditioning, respectively (Grillon and Davis, 1997; Grillon, 2002). In cued fear conditioning, an initially neutral CS is presented such that it temporally predicts the occurrence of an aversive UCS. This association results in “cued” or phasic fear responses upon subsequent presentation of the CS. This contrasts with “contextual” fear responses—non-cue-specific fear of the environment in which an UCS is encountered—which occur when the CS is absent or nonpredictive of UCS occurrence. While multiple mechanisms influence the acquisition and maintenance of cued and contextual fear (Phillips and LeDoux, 1994; Davis and Shi, 1999; Maren and Quirk, 2004; Sotres-Bayon et al., 2004; Phelps et al., 2004; Ji and Maren, 2007), potentially only a subset of these vary substantially in their function across individuals and are linked to differences in trait vulnerability to anxiety. Here, we used functional neuroimaging of cued and contextual fear conditioning in humans to test a *dual-route model of trait vulnerability to anxiety*, according to which two, at least partially, independent dimensions of neurocognitive function are associated with elevated trait vulnerability to anxiety.

We hypothesized that amygdala responsivity to phasic fear cues would provide the first dimension of individual variability associated with trait vulnerability to anxiety, primarily influencing phasic fear acquisition. Here, we built on findings from lesion studies in rodents and neuroimaging research in humans supporting the involvement of the amygdala in the acquisition and expression of cued fear (Maren and Quirk, 2004; Phelps and LeDoux, 2005). This hypothesis was also informed by meta-analyses suggesting that clinically anxious patients show stronger acquisition of cued fear than healthy control participants (Lissek et al., 2005).

Diverging from rodent models of fear conditioning, the second key dimension was predicted to involve the recruitment of ventral prefrontal cortical (vPFC) dependent emotion regulation mechanisms to diminish both cued and contextual fear responses before the omission of threat (i.e., of the UCS). Here, the use of information about CS-UCS contingencies to engage vPFC emotion regulation mechanisms when cued and contextual fear are at their respective peaks is hypothesized to enable individuals to decrease these fear responses prior to extinction. This hypothesis was informed, in part, by models arguing for impoverished contingency-appropriate inhibition of conditioned fear in anxiety (Davis et al., 2000; Lissek et al., 2005) but draws especially upon recent findings indicating that, in humans, vPFC circuitry supports not only the extinction of conditioned fear but also emotion regulation (Ochsner et al., 2002; Ochsner and Gross, 2005; Delgado et al., 2008). The vPFC circuitry underlying emotion regulation encompasses both the medial regions typically implicated in the extinction of conditioned fear and more lateral regions implicated in other forms of cognitive control—e.g., attention regulation (Wager et al., 2008). During intentional emotion regulation, these vPFC regions are coactivated with dorsolateral PFC regions thought to support the deliberate selection of strategies for reappraising emotional stimuli (Ochsner et al., 2002; Mauss et al., 2007; Hartley and Phelps, 2010). The strength of vPFC recruitment during emotion regulation distinguishes individuals able to successfully reduce negative responses to aversive stimuli (Wager et al., 2008). Further, under instruction, emotion regulation can facilitate the reduction of conditioned fear (Delgado et al., 2008). Recently, the role of uninstructed or “automatic” emotion regulation (AER) has received attention, with adaptive forms of AER being proposed to involve recruitment of these same vPFC mechanisms (Mauss et al., 2007). Individual differences in the recruitment of vPFC mechanisms supporting AER might be a key dimension influencing risk for psychopathology. Specifically, we hypothesized that individuals who, without instruction, spontaneously engaged vPFC mechanisms to downregulate acquired fear responses would be less at risk of developing chronic levels of fear and anxiety, with this being reflected in lower trait anxiety scores.

## Results

**Figure 1:**
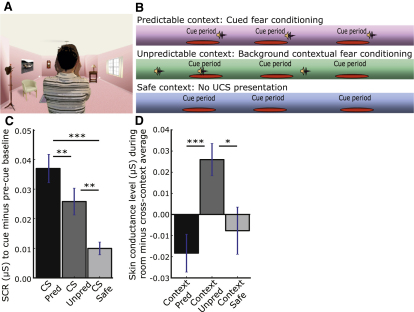
The Cued and Contextual Fear-Conditioning Task (A) An example computerized “room.” The CS takes the form of the virtual actor putting his or her hands to their ears. (B) Schematic illustration of experimental contingencies by room type. *Cue period* = time from CS onset to CS offset. Sound symbol = UCS (750 ms 103 dB scream). (C) Skin conductance response (SCR) during early acquisition to CS relative to pre CS baseline by room type. (D) Skin conductance level during early acquisition for presentation of each room relative to mean across rooms. ^∗^p < 0.05, ^∗∗^p < 0.01, ^∗∗∗^p < 0.001, all one-tailed, data are natural log transformed. Error bars represent SEM.

In order to test these predictions, we administered a functional neuroimaging cued and contextual fear conditioning task to healthy young volunteers with varying levels of trait anxiety, as measured by the trait subscale of the Spielberger State-Trait Anxiety Inventory, Form Y (Spielberger, 1983) (see Experimental Procedures). We manipulated CS-UCS contingencies within different visual environments in order to investigate the engagement of amygdala and vPFC mechanisms during conditions promoting cued and contextual fear as a function of trait anxiety. Given that our normal world involves complex multifeatured environments, changing a single property, such as background screen color, may not suffice for contextual manipulation of conditioned fear (Grillon, 2008). Consequently, we constructed three alternate computerized “rooms” complete with different items of furniture as well as different color schemes (Figure 1A). The CS was a virtual actor putting their hands to their ears, the UCS being a 750 ms 103 dB scream. In the “predictable” (cued fear) room, CS offset was always accompanied by UCS presentation. In the “unpredictable” (contextual fear) room, CS and UCS presentation were unpaired. In a third “safe” room, CS presentation occurred without UCS presentation (Figure 1B) (see Experimental Procedures for further details). Prior findings suggest that anxiety may influence the extent to which partially predictive cues give rise to cued versus contextual fear (Tsetsenis et al., 2007; Baas et al., 2008). Hence to maximize differentiation of our cued and contextual fear conditions, we used a 100% CS-UCS contingency in the predictable (cued fear) room, with CS duration being varied to enable separation of the blood oxygen level dependent (BOLD) response to CS onset versus UCS onset. Amygdala involvement in cued fear was assessed by comparison of the amygdala response to this predictive CS relative to the CS in the “safe” room. This contrast is held to best illuminate the mechanisms underlying cued or phasic fear, without influence by potential anxiety-related differences in the processing of cues presented in a nonpredictive relationship with the UCS (Lissek et al., 2005). Context-appropriate recruitment of vPFC mechanisms to downregulate both cued and contextual fear responses, at their respective peaks, was assessed by examining phasic (CS-specific) and sustained (throughout room presentation) vPFC activity for the predictable (cued fear) versus unpredictable (contextual fear) conditions.

All participants completed an initial 10 min early-acquisition/training session of the fear-conditioning task outside of the scanner and then, 48 hr later, completed four further 8 min runs while functional magnetic resonance imaging (fMRI) data were acquired. Galvanic skin conductance was measured during both sessions. Cross-group analysis of these data supported the stronger acquisition of cued fear responses in the predictable room and of contextual fear (sustained throughout presentation of the context) in the unpredictable room (Figures 1C and 1D; see Figure S1 available online).

### Amygdala Responsivity to Phasic Fear Cues

**Figure 2:**
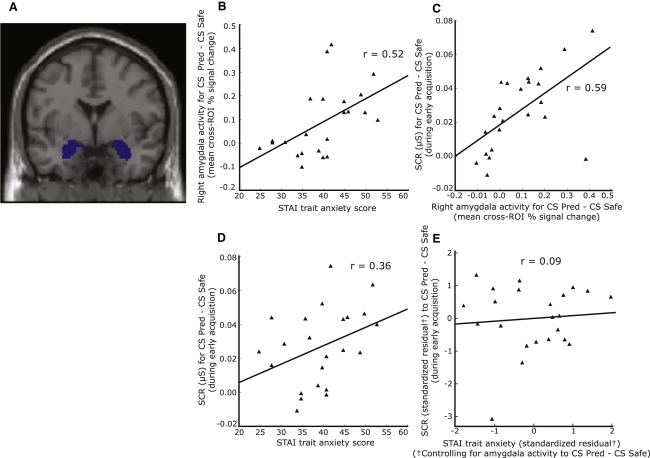
Individuals High in Trait Anxiety Showed Increased Amygdala Responsivity to Phasic Fear Cues; This Was Linked to Stronger Initial SCR Acquisition to These Cues (A) Amygdala ROIs were defined using the Montreal Neurological Institute (MNI) template Automated Anatomical Labeling (AAL) map. (B) There was a significant positive correlation between trait anxiety and right amygdala activity^‡^ to cues that predicted UCS occurrence (CS Pred) relative to cues that occurred in the absence of UCS presentation (CS Safe). (C) Amygdala activity to the predictive CS versus the safe room CS was significantly correlated with the strength of early phasic fear acquisition (SCR to predictive CS versus safe room CS during the initial acquisition session). (D) Individuals with higher trait anxiety scores showed significantly stronger early phasic fear acquisition. (E) With individual differences in amygdala activity to the predictive CS versus the safe room CS controlled for, the relationship between trait anxiety and early phasic fear acquisition was no longer significant. ^‡^Note: This was extracted and averaged across the ROI to avoid peak voxel inflation of the correlation. A similar trend was observed for the left amygdala ROI, r(21) = 0.30, p = 0.08, activation in this ROI also showing a strong relationship with initial SCR acquisition to the predictive (versus safe) CS, r(21) = 0.46, p < 0.05.

The first hypothesis we tested was that individuals with elevated trait anxiety would show hyperresponsivity of amygdaloid mechanisms involved in the acquisition and expression of phasic (cued) fear. In order to address this, we examined participants' amygdala response to the CS in the predictable room (CSpred) relative to that in the safe room (CSsafe) as a function of trait anxiety. The BOLD signal associated with the CSpred-CSsafe contrast was extracted and averaged across all voxels within bilateral amygdala regions of interest (ROIs, as shown in Figure 2A) on a subject by subject basis (see Experimental Procedures). This composite measure of amygdala activity was then correlated against participant trait anxiety, avoiding the issues arising from peak voxel statistics associated with small volume corrected search based techniques (Vul et al., 2009). In line with our predictions, trait anxiety was positively correlated with the magnitude of the amygdala response to presentation of the predictive CS versus the safe CS, r(21) = 0.52, p < 0.01 (Figure 2B); the association remaining significant after the effects of state anxiety were controlled for, r(20) = 0.43, p < 0.05. This index of cued fear associated amygdala activity was in turn significantly correlated with the strength of initial cued fear acquisition, as measured by the skin conductance response (SCR) to the predictive CS versus the safe CS during the early acquisition/training session, r(21) = 0.59, p < 0.005 (Figure 2C). Trait anxiety was also positively associated with this SCR measure of initial cued fear acquisition, r(21) = 0.36, p < 0.05 (Figure 2D). Results from a mediation analysis were consistent with the relationship between trait anxiety and initial cued fear acquisition (SCR to CSpred versus CSsafe) being mediated by differences in amygdala responsivity to phasic fear cues (CSpred versus CSsafe), Soebel test statistic = 1.90, p < 0.05. With the variance attributable to individual differences in amygdala responsivity to phasic fear cues controlled for, the relationship between trait anxiety and strength of initial cued fear acquisition no longer reached significance, r(20) = 0.09, p > 0.3 (Figure 2E). There was no significant relationship between trait anxiety and the amygdala response to the nonpredictive CS relative to the safe CS, p > 0.1. These findings support the contention that individual differences in amygdala responsivity to phasic fear cues provide one dimension of neurocognitive function through which trait vulnerability to anxiety may confer risk for development of pathological fear responses—in particular, the acquisition of cue-specific fears characteristic of conditions such as specific phobia.

### Recruitment of vPFC Mechanisms to Downregulate Cued and Contextual Fear

**Figure 3:**
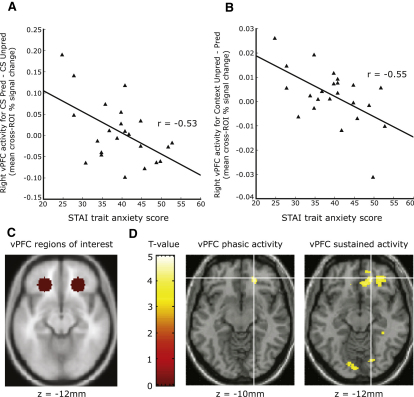
Individuals High in Trait Anxiety Showed Impoverished Pre-extinction Ventral Prefrontal Cortical (vPFC) Recruitment at Points Where Cued and Contextual Fear Responses Were, Respectively, at Their Peaks (A) Elevated trait anxiety was associated with a reduced phasic vPFC response to cues predictive of UCS occurrence (relative to nonpredictive cues). (B) High trait anxious individuals also showed reduced sustained vPFC activity throughout presentation of the “unpredictable” context in which CS presentation did not predict UCS occurrence (versus throughout presentation of the “predictable” context). (C) Regions of interest across which vPFC activity was extracted and averaged (taken from Wager et al. [2004], as detailed in Experimental Procedures). (D) Confirmatory voxel-wise analyses show the peak (cross-hairs) and extent (thresholded at t > = 3.5) of the phasic and sustained vPFC responses.

The second hypothesis we tested was that high trait anxious individuals would show weaker contingency-appropriate recruitment of vPFC regulatory mechanisms at points of maximal cued and contextual fear prior to UCS omission. We thus examined whether heightened trait anxiety was associated both with a reduced phasic vPFC response to presentation of the predictive CS versus nonpredictive CS and with weaker sustained vPFC recruitment throughout presentation of the unpredictable room relative to the predictable room. Our data provided evidence in support of this, with high trait anxious individuals showing reduced phasic vPFC recruitment in response to the predictive CS, r(21) = –0.53, p < 0.005, and weaker sustained vPFC activity during the unpredictable room, r(21) = –0.55, p < 0.005 (Figures 3A and 3B). Both of these relationships remained significant after the effects of state anxiety were controlled for, r(20) = –0.46, p < 0.02, r(20) = –0.52, p < 0.01, respectively. The magnitude of the phasic vPFC response was inversely correlated with the concurrent (i.e., during scan session) SCR to the predictive CS (versus the nonpredictive CS), r(21) = –0.60, p < 0.002 (Figure S3A), in line with our contention that contingency-sensitive recruitment of vPFC mechanisms aids in the downregulation of conditioned fear prior to UCS omission. In addition, those individuals showing higher sustained vPFC recruitment across the unpredictable room also showed lower concurrent skin conductance levels throughout presentation of this room, r(21) = –0.47, p < 0.02 (with respect to the “safe” room baseline) (Figure S3B). The vPFC response to cued fear (CSpred versus CS unpred) as modulated by trait anxiety was strongly right-lateralized, Williams-Hotelling t(20) = 2.51, p < 0.05. In the case of the contextual fear contrast, the vPFC response modulated by trait anxiety was less lateralized, Williams-Hotelling, t(20) = 1.07, p > 0.1, there also being a trend toward trait anxiety being associated with reduced sustained left vPFC recruitment throughout presentation of the unpredictable room relative to the predictable room, r(21) = –0.29, p = 0.09, one-tailed. Confirmatory whole-brain voxel-wise analyses revealed that while the region of right vPFC phasically activated to downregulate the cued fear response to the predictive CS was relatively constrained, the sustained vPFC response to contextual fear—during the unpredictable room—was of greater spatial extent, spreading from a focal point within our a-priori ROI (as detailed in the Experimental Procedures) to encompass both medial and lateral vPFC regions (Figures 3C and 3D).

### Two Independent Dimensions of Trait Vulnerability to Anxiety?

In order to test the prediction that (1) increased amygdala responsivity to phasic fear cues and (2) impoverished CS-UCS contingency-appropriate recruitment of vPFC regulatory mechanisms are independently related to trait vulnerability to anxiety, we conducted hierarchical regression analyses with trait anxiety as the dependent variable. The three neural indices of interest—the phasic amygdala response to the predictive CS versus safe CS, the phasic vPFC response to the predictive CS versus the nonpredictive CS, and sustained vPFC activity across the unpredictable room versus the predictable room—were considered as predictors. Significantly greater variance in trait anxiety scores was accounted for by including both the amygdala response to the predictive CS versus safe CS and either of the vPFC indices as predictors than by entry of any one of these variables alone (*P*s < 0.05). This is consistent with amygdala responsivity to phasic fear cues providing one dimension of neurocognitive function associated with trait vulnerability to anxiety and with a second dimension comprising contingency-appropriate phasic and sustained recruitment of vPFC regulatory mechanisms. The optimal model was given by entry of the amygdala and phasic vPFC indices, both of these measures contributing independently to prediction of trait anxiety scores (amygdala β = 0.55, p < 0.002, phasic vPFC β = –0.56, p < 0.001) (see Table 1). No additional benefit was derived from entering the sustained vPFC measure once the phasic vPFC response was in the model (ΔR^2^ = 0.02, p > 0.1), nor from entering any of the interaction terms for the three predictor variables, *Ps* > 0.1.

There was a negative zero-order correlation between vPFC activity to the predictive CS versus safe CS and trait anxiety, r(21) = –0.42, p < 0.05, and a trend toward a positive zero-order correlation between amygdala activity to the predictive CS versus nonpredictive CS and trait anxiety, r(21) = 0.34, p = 0.06. Additional regression analyses revealed that the optimal model detailed above was not changed by inclusion of these indices as predictor variables nor by inclusion of regressors reflecting amygdala and vPFC activity associated with any of the other cue (CS) or context (room) related contrasts (*P*s > 0.1).

### Hippocampal-vPFC Corecruitment

In addition to amygdaloid and vPFC dysregulation, disruption to hippocampal-dependent mechanisms has also been posited as a potential source of dysfunction in anxiety (e.g., Tsetsenis et al., 2007). The hippocampus is thought to have an important role in the contextual modulation of conditioned fear. The extinction of conditioned fear has been shown to be especially sensitive to contextual influences, with hippocampal-vPFC interactions being held to facilitate context-specific extinction of conditioned fear (Sotres-Bayon et al., 2004; Ji and Maren, 2007). Results from our current study suggest that high trait anxious individuals show an impoverished ability to recruit vPFC regions in a context-selective phasic or sustained manner to downregulate cued and contextual fear, prior to extinction. This raises the question of whether either impaired hippocampal function or disruption to hippocampal-vPFC connectivity might contribute to high trait anxious individuals' difficulties with context appropriate engagement of vPFC mechanisms to facilitate the downregulation of conditioned fear.

**Figure 4:**
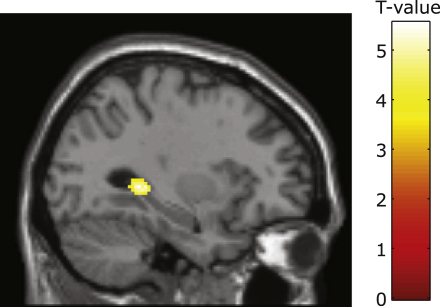
High Trait Anxiety Was Associated with Reduced Connectivity between Right vPFC and Bilateral Hippocampal Regions across Contexts This is shown for the left hippocampus, here the negative correlation between trait anxiety and right vPFC–hippocampal connectivity survived both small volume correction using the MNI AAL hippocampal ROI (p < 0.005) and whole brain cluster level correction (p < 0.05). Voxels showing reduced connectivity to the right vPFC seed region as a function of trait anxiety are displayed, superimposed on the SPM 5 canonical single subject T1 structural. At a whole-brain threshold of p < 0.001 uncorrected, posterior hippocampus is the predominant region showing reduced connectivity to the right vPFC seed region as a function of trait anxiety. A similar effect of trait anxiety upon left vPFC-hippocampal coupling was observed, p = 0.001 uncorrected, but this did not survive correction for multiple comparisons. Hierarchical regression analyses revealed that neither phasic nor sustained indices of hippocampal activity contributed significantly to prediction of trait anxiety scores once amygdala and vPFC regressors were included as predictor variables. Plots showing zero-order correlations between hippocampal activation indices and trait anxiety are given in Figure S4.

In order to examine this, we extended our regression analyses to include measures of hippocampal activity associated with each of our contrasts of interest, and additionally investigated modulation by trait anxiety of functional connectivity between our vPFC ROIs and the hippocampus. There was a zero-order positive correlation between hippocampal activity to the nonpredictive CS versus the safe room CS and trait anxiety, r(21) = 0.53, p < 0.01, and a nonsignificant trend toward a negative relationship between sustained hippocampal activity during the unpredictable room versus the predictable room and trait anxiety, r(21) = –0.32, p = 0.14, 2-tailed. However, hierarchical regression analyses indicated that neither entry of these nor any other index of hippocampal activity altered the model that best predicted trait anxiety scores, this remaining as described above. Specifically, with indices of amygdala and vPFC function included as predictor variables, the relationship between hippocampal function and trait anxiety was not significant (*P*s > 0.1). In order to examine whether trait anxiety modulated vPFC-hippocampal co-recruitment, we conducted functional connectivity analyses, using vPFC as the seed region (see Experimental Procedures for further details). These revealed that elevated trait anxiety was significantly associated with reduced vPFC-hippocampal connectivity across contexts (Figure 4).

## Discussion

The results presented here provide insight into the mechanisms by which trait vulnerability to anxiety may increase risk of pathological fear and anxiety responses. Our data indicate that there are two dimensions of variability in neurocognitive function associated with trait vulnerability to, versus resilience from, anxiety. First, high trait anxious individuals showed increased amygdala responsivity to cues that predicted the occurrence of an aversive stimulus. The magnitude of this response was associated with the strength of initial cued fear acquisition. This raises the possibility that individual variability in amygdala responsivity to cues or objects temporally paired with aversive events might contribute to differences in vulnerability to anxiety disorders with a strong phasic fear component.

Our data further suggest that individual variability in context-appropriate recruitment of vPFC mechanisms to downregulate cued and contextual fear prior to UCS omission may provide a second important dimension through which trait vulnerability to, versus resilience from, anxiety is conferred. In line with both rodent and human fear-conditioning literatures (Odling-Smee, 1975; Grillon and Davis, 1997), presentation of cues that predicted UCS occurrence led to phasic increases in skin conductance (“cued fear”), while contexts in which cues were nonpredictive of UCS occurrence were associated with sustained elevation of skin conductance levels (“contextual fear”). Low trait anxious individuals showed both increased phasic vPFC recruitment to cues that predicted the UCS and increased sustained vPFC recruitment across contexts in which UCS occurrence was unpredictable. The strength of these phasic and sustained vPFC signals, respectively, was inversely associated with concurrent skin conductance indices of cued and contextual fear expression. Such contingency-appropriate recruitment of vPFC mechanisms to downregulate conditioned fear responses *prior* to the omission of the UCS might confer resilience against the development of pathological fear and anxiety, especially in times of ongoing adversity. Conversely, the reduced engagement of these mechanisms by high trait anxious individuals could contribute to the maintenance of symptoms of both phasic fear and nonspecific anxiety.

Our hierarchical regression analyses suggest that amygdala responsivity to phasic fear cues and impoverished contingency-appropriate recruitment of vPFC regulatory mechanisms are independently associated with trait vulnerability to anxiety. Differential dysregulation of these mechanisms could potentially account for variability in phasic fear and nonspecific anxiety symptomatology across different anxiety disorders. Heritability studies suggest that two common genetic factors may differentially increase risk for various anxiety disorders, one factor loading heavily on disorders characterized by cue-specific fear (e.g., specific phobia), but both contributing strongly to conditions such as generalized anxiety disorder (Hettema et al., 2006). Our current findings provide evidence for a parallel dual-route model at a neurocognitive level of analysis. Dysregulation of both the amygdala and vPFC mechanisms identified here may influence the strength of phasic fear responses, disruption to the latter potentially also underlying persistent symptoms of nonspecific anxiety.

With amygdala and vPFC factors in the regression model, neither phasic nor sustained hippocampal activity indices contributed a third independent dimension associated with trait vulnerability to anxiety. However, functional connectivity analyses indicated that high trait anxious individuals showed reduced vPFC-hippocampal connectivity. This is consistent with hippocampal mechanisms being involved in context-selective phasic versus sustained activation of vPFC to downregulate cued and contextual fear prior to omission of the UCS, and with disrupted interactions between the hippocampus and vPFC potentially contributing to the reduced context-appropriate recruitment of vPFC regulatory mechanisms shown by high trait anxious individuals. This falls in line with findings from prior research with both animals and humans that have suggested a role for the hippocampus in the contextual control of conditioned fear extinction and extinction recall (Kalisch et al., 2006; Ji and Maren, 2007; Milad et al., 2007; Lang et al., 2009).

An understanding of the neurocognitive mechanisms by which trait vulnerability to pathological anxiety is conferred may aid, not only in explaining variability in symptomatology across disorders, but also in informing choice of intervention, and prediction of treatment response. With regards to intervention, our finding that low trait anxious individuals recruited vPFC mechanisms to decrease both cued and contextual fear before UCS omission is of particular interest. The activation, in humans, of vPFC regions during both fear extinction and emotion regulation has been held to represent the adoption of phylogenetically old mechanisms of extinction to facilitate new means of reducing nonadaptive emotional responses (Hartley and Phelps, 2010). Our data suggest that these mechanisms may be *spontaneously* engaged by low trait anxious individuals to downregulate conditioned fear. The instructed use of emotion regulation techniques to reduce phasic fear responses has been demonstrated in nonanxious volunteers (Delgado et al., 2008). An important question for future work is whether trait anxiety-related deficits in the apparently spontaneous recruitment of vPFC mechanisms to diminish cued and contextual fear could be remediated by training in deliberate emotion regulation techniques.

## Experimental Procedures

### Participants and Anxiety Measurement

Twenty-three participants (13 females, 10 males, right-handed, aged 18–41 years, mean age = 25 years), performed a fear-conditioning task while functional magnetic resonance imaging (fMRI) and galvanic skin conductance data were acquired. The study was approved by the Cambridgeshire Local Research Ethics Committee and carried out in compliance with their guidelines. Written informed consent was obtained from all participants. Individuals with a history of psychiatric care, neurological disease or head injury were excluded, as were individuals on medication for anxiety or depression. Trait and state anxiety were measured using the Spielberger State-Trait Anxiety Inventory, Form Y (STAI; Spielberger, 1983). This provides a widely used measure of trait vulnerability to anxiety. Scores on the trait subscale are elevated in individuals who meet criteria for anxiety disorders, across subtypes (Bieling et al., 1998; Chambers et al., 2004). In addition, elevated STAI trait anxiety scores have been shown to predict future AD diagnosis (Plehn and Peterson, 2002; Chambers et al., 2004). Unlike other self-report measures, the STAI enables investigation of the correlates of trait anxiety, while controlling for between-participant differences in state anxiety. It should be noted that STAI trait anxiety scores are also elevated in individuals with major depressive disorder (MDD), potentially related to the strong shared symptomatology, and heritability, of generalized anxiety disorder and MDD (Chambers et al., 2004). Prior to the initial training session, participants completed the STAI trait and state anxiety subscales. At the beginning of the fMRI session they were readministered the state subscale. Participants' state anxiety scores ranged from 20 to 43 prior to training (mean = 32, SD = 7) and from 21 to 54 (mean = 33, SD = 8) at the beginning of the fMRI session. Trait anxiety scores ranged from 25 to 53 (mean = 40, SD = 8). These scores are comparable to the published norms for this age group (Spielberger, 1983).

### Stimuli and Procedure

Visual stimuli were developed in Matlab 7.1 using Cogent 2000 1.25 software (MathWorks Inc., Natick, MA). The fear-conditioning task examined cued fear-conditioning and background contextual conditioning. Three different computerized environments or “rooms” were created to manipulate context. These varied both in predominant color (pink, green, blue) and in furniture (Figure 1A). The contingency between the conditioned stimulus (CS) and presentation of the unconditioned stimulus (UCS) differed between rooms. In the “predictable” (cued fear) room, the CS was predictive of the UCS; in the “unpredictable” (background contextual fear) room, the CS was nonpredictive of UCS occurrence, and in the “safe” (control) room, CS presentation occurred in the absence of the UCS. Each room was presented for approximately 40 s. The CS was a virtual actor (male or female) putting hands to ears as if to protect him- or herself from a loud sound. This gesture terminated after 4–6 s. In the predictable room, CS offset was always accompanied by UCS presentation. The UCS was a 103 dB scream lasting 750 ms. In the unpredictable room, UCS presentation was randomized with regards to CS presentation. Each predictable and unpredictable room presentation contained three CS and three UCS occurrences. Each safe room presentation contained three CS occurrences.

In order to ensure participants stayed engaged, we added a behavioral component to the task. During each room presentation, the virtual actor would occasionally turn around. Participants were instructed to push a button with the right index finger in response to this. These behavioral responses were not analyzed, except so far as to ensure continued attention to the task (i.e., that the volunteer was awake, alert, and making responses).

Each participant was trained on the fear-conditioning task 48 hr before the scanning session. During this initial session, participants completed one run comprising five repetitions of each room, presented in a randomized order, while skin conductance was recorded. At the end of the session we asked participants to rank the rooms from the most liked to the less preferred, with an explanation for their preference. All participants indicated awareness of the CS-UCS contingencies associated with each room.

During the scan session, we presented four task runs each comprising four repetitions of each room type, presented in a randomized order. Visual stimuli were back projected onto a translucent screen positioned behind the bore of the magnet and were viewed via an angled mirror placed above the participant's head. FMRI data were acquired and skin conductance measured during task performance.

### fMRI Data Acquisition

Blood oxygenation level dependent (BOLD) contrast functional images were acquired with echo-planar T2^∗^-weighted (EPI) imaging using a Siemens Tim Trio 3T MR system with a 12 channel head coil. Each image volume consisted of 48 interleaved 2-mm-thick slices (interslice gap, 0.5mm; inplane resolution, 3^∗^3 mm; flip angle, 90°; echo time, 30 ms; bandwidth, 2232 Hz; repetition time, 3.0 s). Slice acquisition was transverse oblique, angled to avoid the eyeballs, and covered the whole brain. Data were acquired in four scanning runs of 8 min. The first six volumes of each run were discarded to allow for T1 equilibration effects. T1-weighted structural images were acquired at a resolution of 1 × 1 × 1 mm.

### fMRI Data Analysis

FMRI data were analyzed using Matlab version 7.3 and Statistical Parametric Mapping (SPM) version5 software (Wellcome Department of Imaging Neuroscience, London, UK). We conducted standard preprocessing, including realignment, to correct for head movement, and normalization of each participant's EPI data to the Montreal Neurological Institute International Consortium for Brain Mapping (MNI) template (Tzourio-Mazoyer et al., 2002). Images were resampled into this space with 2 mm isotropic voxels. A high-pass filter of 260 s was used to remove low-frequency noise.

A mixed-model design was used (Visscher et al., 2003). Events were modeled by step functions convolved with the canonical hemodynamic response function (HRF) to form regressors. The onset for each type of CS (CS from each room) was modeled with a separate regressor. The UCS (scream events) was modeled using a single regressor across rooms to facilitate separation of the BOLD response to the UCS from that to the CSs. This was also facilitated by varying CS duration. This enabled the period between predictive CS onset and UCS onset to be jittered despite cotermination of this CS with UCS presentation. The three “rooms” or contexts were modeled by step functions with the duration for which the given room was presented (∼40 s). These were also convolved with the HRF to form regressors. Motion parameters were included in the design matrix as covariates of no interest.

At the single subject level of analysis, the MarsBar ROI toolbox (http://marsbar.sourceforge.net) (Brett et al., 2002) was used to extract the mean activity (across voxels) associated with each contrast of interest from our a priori regions of interest (ROIs). This was conducted using normalized but nonsmoothed data. For the amygdala and hippocampus, we used bilateral ROIs defined by the MNI template Automated Anatomical Labeling (AAL) map. For ventral prefrontal cortex (vPFC), we used functionally defined regions. Given issues recently raised in the literature regarding the nonindependence of ROI selection from subsequent analyses (Vul et al., 2009), in order to ensure avoidance of bias, we adopted ROIs previously used by our group in a study of the regulation of emotional processing (Bishop et al., 2006). These consisted of 10mm radius spheres centered on coordinates (x,y,z = ±24 34 –12) derived from activations initially reported in a study of expectation of aversive stimuli (Wager et al., 2004). Left and right ROIs were analyzed independently. This decision was informed by both methodological and theoretical considerations including the debate within the neuropsychological literature as to the privileged role of right, versus left, vPFC in emotion regulation (Tranel et al., 2002; see also Wager et al., 2008).

A random effects analysis was used to analyze data at a group level, with effects of trait anxiety being assessed by regression of ROI mean activity associated with a given contrast against trait anxiety scores from the STAI. This approach, as opposed to voxel-wise analyses small volume corrected for multiple comparisons, was adopted in order to avoid peak-voxel inflation of correlation estimates (Vul et al., 2009). Contrasts of interest included comparisons of sustained activity across rooms (“unpredictable”/ background contextual fear room versus “predictable”/ cued fear room and “safe”/ control room) and phasic activity to CS onset as a function of room type (e.g., to the predictive CS in the cued fear room versus to the nonpredictive CS in the background contextual fear room). Confirmatory voxel-wise whole-brain analyses were conducted to provide information as to the spread of activation captured by these ROI-based analyses (for these whole-brain voxel-wise analyses the data was smoothed with a Gaussian kernel of 10 mm full-width at half-maximum). In addition, all of the trait-anxiety analyses reported here were repeated substituting STAI trait anxiety scores with scores from the STAI state anxiety subscale. None of these state anxiety correlations reached significance (*P*s > 0.1, two-tailed).

Functional connectivity analyses were conducted to examine vPFC-hippocampal connectivity, using right and left vPFC ROIs, separately, as seed regions. An empirical Bayesian deconvolution algorithm (SPM 5, PsychoPhysiological Interaction software) (Friston et al., 1997; Gitelman et al., 2003) was employed to obtain a deconvolved fMRI time series from the vPFC ROIs for each subject. This physiological regressor, the block regressor for each room type, and the product of the physiological regressor and each block regressor were entered into a new model and reconvolved with the HRF. Movement parameters were also entered. Contrasts from this model were taken forward to a random effects analysis where trait anxiety scores were entered as a covariate of interest enabling a voxel-wise investigation of regions showing increased or decreased connectivity with vPFC as a function of trait anxiety.

Supplementary finite impulse response (FIR) analyses were also conducted in order to examine the time course of phasic amygdala and vPFC responses to CS onset as a function of room type and the modulation of activity for each FIR timebin by individual differences in trait anxiety (see Supplemental Experimental Procedures, Figure S2, and Table S1 for further details).

### Galvanic Skin Conductance Data Acquisition

A Biopac MP 150 System together with Acknowledge software (Biopac Inc., Goleta, CA) was used to record skin conductance data during the initial training session and during task performance within the MRI environment. Two Ag-AgCl electrodes spread with electrolyte paste were positioned on the palm of the left hand. These were connected to a Biopac GSR100C module with the gain set to 5 microSiemens/V, the low pass filter to 1.0 Hz, and the high pass filters to DC. For the scan session, an MRI-compatible version of the equipment was used (http://www.biopac.com/Manuals/mecmri.pdf). A continuous skin conductance signal was output into Acknowledge 3.9 software on an analysis computer and time-stamped to indicate the onset and offset of each event by means of digital markers sent from the stimulus delivery computer. Data were acquired at 200 samples per second. The data was transformed into microsiemens (μS) before being analyzed.

### Galvanic Skin Conductance Data Analysis

Due to nonnormality of the data, a natural log (x+1) transform was applied to the raw data from both the training and scan sessions for each participant. The skin conductance response (SCR) to CS presentation was assessed as the base to peak difference with baseline being estimated using the mean signal across the 2 s period immediately prior to CS onset and the peak response being extracted from the period between CS onset and offset (Orr et al., 2000; Milad et al., 2005, 2007). In the training session, the mean SCR was calculated for each CS as a function of room type (i.e., for CSpred, CSunpred, CSsafe). The scan SCR data remained skewed after log transformation. Given this, within each run the median SCR was calculated for each CS. The median values from each of the four runs were then averaged to give a final estimate of the SCR for each CS type. Mean skin conductance levels (SCL) were also obtained for each room presentation. The median value within a given run was calculated—median being used to reduce the impact of outlying data points. For the scan session, the resultant values were averaged across runs. Following this the mean of the three room type SCL scores was subtracted in order to give an estimate of SCL by room type relative to the subject's average SCL during the session in question.

## Figures and Tables

**Table 1:** Results from Hierarchical Linear Regression Analyses with Trait Anxiety as the Dependent Variable and Indices of Task-Related Regional Neural Activity as Predictors

Model	No. of Predictors	R^2^	Sig.	Change in (Δ) R^2^	Significance of ΔR^2^
Amygdala	1	0.27	0.012^∗^	**-**	**-**
s-vPFC	1	0.31	0.006^∗∗^	**-**	**-**
p-vPFC	1	0.28	0.010^∗∗^	**-**	**-**
Amygdala, s-vPFC	2	0.46	0.002^∗∗∗^	0.19 (s-vPFC adj Amygdala), 0.15 (Amygdala adj s-vPFC)	0.015^∗^, 0.029^∗^
**Amygdala,** **p-vPFC**	**2**	**0.58**	**0.000^∗∗∗∗^**	**0.30 (Amygdala adj p-vPFC), 0.31 (p-vPFC adj Amygdala)**	**0.002^∗∗∗^,** **0.001^∗∗∗∗^**
s-vPFC, p-vPFC	2	0.38	0.009^∗∗^	0.10 (s-vPFC adj p-vPFC), 0.07 (p-vPFC adj s-vPFC)	n.s., n.s.

^∗^p < 0.05, ^∗∗^p < 0.01, ^∗∗∗^p < 0.005, ^∗∗∗∗^p < 0.001. Predictor variables: phasic amygdala response to the predictive CS versus safe CS (amygdala), phasic vPFC response to the predictive CS versus the nonpredictive CS (p-vPFC), sustained vPFC activity across the unpredictable room versus the predictable room (s-vPFC). The optimal model is shown in boldface.

## Supplemental Information

Document S1. Supplemental Experimental Procedures, Four Figures, and One Table

## References

[bib1] Baas J.M., van Ooijen L., Goudriaan A., Kenemans J.L. (2008). Failure to condition to a cue is associated with sustained contextual fear. Acta Psychol. (Amst.).

[bib2] Bieling P.J., Antony M.M., Swinson R.P. (1998). The state-trait anxiety inventory, trait version: Structure and content re-examined. Behav. Res. Ther..

[bib3] Bishop S.J. (2007). Neurocognitive mechanisms of anxiety: An integrative account. Trends Cogn. Sci..

[bib4] Bishop S.J., Cohen J.D., Fossella J., Casey B.J., Farah M.J. (2006). COMT genotype influences prefrontal response to emotional distraction. Cogn. Affect. Behav. Neurosci..

[bib5] Brett M., Anton J., Valabregue R., Poline J. (2002). Region of interest analysis using an SPM toolbox. Neuroimage.

[bib6] Chambers J.A., Power K.G., Durham R.C. (2004). The relationship between trait vulnerability and anxiety and depressive diagnoses at long-term follow-up of generalized anxiety disorder. J. Anxiety Disord..

[bib7] Davis M., Shi C. (1999). The extended amygdala: Are the central nucleus of the amygdala and the bed nucleus of the stria terminalis differentially involved in fear versus anxiety?. Ann. N.Y. Acad. Sci..

[bib8] Davis M., Falls W.A., Gewirtz J., Myslobodsky M., Weiner I. (2000). Neural systems involved in fear inhibition: Extinction and conditioned inhibition. Contemporary Issues in Modeling Psychopathology.

[bib9] Delgado M.R., Nearing K.I., Ledoux J.E., Phelps E.A. (2008). Neural circuitry underlying the regulation of conditioned fear and its relation to extinction. Neuron.

[bib10] Friston K.J., Buechel C., Fink G.R., Morris J., Rolls E., Dolan R.J. (1997). Psychophysiological and modulatory interactions in neuroimaging. Neuroimage.

[bib11] Gitelman D.R., Penny W.D., Ashburner J., Friston K.J. (2003). Modeling regional and psychophysiologic interactions in fMRI: The importance of hemodynamic deconvolution. Neuroimage.

[bib12] Grillon C. (2002). Startle reactivity and anxiety disorders: Aversive conditioning, context, and neurobiology. Biol. Psychiatry.

[bib13] Grillon C. (2008). Models and mechanisms of anxiety: Evidence from startle studies. Psychopharmacology (Berl.).

[bib14] Grillon C., Davis M. (1997). Fear-potentiated startle conditioning in humans: Explicit and contextual cue conditioning following paired versus unpaired training. Psychophysiology.

[bib15] Hartley C.A., Phelps E.A. (2010). Changing fear: The neurocircuitry of emotion regulation. Neuropsychopharmacology.

[bib16] Hettema J.M., Neale M.C., Myers J.M., Prescott C.A., Kendler K.S. (2006). A population-based twin study of the relationship between neuroticism and internalizing disorders. Am. J. Psychiatry.

[bib17] Ji J., Maren S. (2007). Hippocampal involvement in contextual modulation of fear extinction. Hippocampus.

[bib18] Kalisch R., Korenfeld E., Stephan K.E., Weiskopf N., Seymour B., Dolan R.J. (2006). Context-dependent human extinction memory is mediated by a ventromedial prefrontal and hippocampal network. J. Neurosci..

[bib19] Lang S. (2009). Context conditioning and extinction in humans: Differential contribution of the hippocampus, amygdala and prefrontal cortex. Eur. J. Neurosci..

[bib20] Lissek S. (2005). Classical fear conditioning in the anxiety disorders: A meta-analysis. Behav. Res. Ther..

[bib21] Maren S., Quirk G.J. (2004). Neuronal signaling of fear memory. Nat. Rev. Neurosci..

[bib22] Mauss I.B., Bunge S.A., Gross J.J. (2007). Automatic emotion regulation. Soc. Pers. Psychol. Compass.

[bib23] Milad M.R., Orr S.P., Pitman R.K., Rauch S.L. (2005). Context modulation of memory for fear extinction in humans. Psychophysiology.

[bib24] Milad M.R., Wright C.I., Orr S.P., Pitman R.K., Quirk G.J., Rauch S.L. (2007). Recall of fear extinction in humans activates the ventromedial prefrontal cortex and hippocampus in concert. Biol. Psychiatry.

[bib25] Mineka S., Oehlberg K. (2008). The relevance of recent developments in classical conditioning to understanding the etiology and maintenance of anxiety disorders. Acta Psychol. (Amst.).

[bib26] Ochsner K.N., Gross J.J. (2005). The cognitive control of emotion. Trends Cogn. Sci..

[bib27] Ochsner K.N., Bunge S.A., Gross J.J., Gabrieli J.D. (2002). Rethinking feelings: An FMRI study of the cognitive regulation of emotion. J. Cogn. Neurosci..

[bib28] Odling-Smee F.J. (1975). The role of background stimuli during Pavlovian conditioning. Q. J. Exp. Psychol..

[bib29] Orr S.P., Metzger L.J., Lasko N.B., Macklin M.L., Peri T., Pitman R.K. (2000). D. novo conditioning in trauma-exposed individuals with and without posttraumatic stress disorder. J. Abnorm. Psychol..

[bib30] Phelps E.A., LeDoux J.E. (2005). Contributions of the amygdala to emotion processing: From animal models to human behavior. Neuron.

[bib31] Phelps E.A., Delgado M.R., Nearing K.I., LeDoux J.E. (2004). Extinction learning in humans: Role of the amygdala and vmPFC. Neuron.

[bib32] Phillips R.G., LeDoux J.E. (1994). Lesions of the dorsal hippocampal formation interfere with background but not foreground contextual fear conditioning. Learn. Mem..

[bib33] Plehn K., Peterson R.A. (2002). Anxiety sensitivity as a predictor of the development of panic symptoms, panic attacks, and panic disorder: A prospective study. J. Anxiety Disord..

[bib34] Quirk G.J., Garcia R., González-Lima F. (2006). Prefrontal mechanisms in extinction of conditioned fear. Biol. Psychiatry.

[bib35] Sotres-Bayon F., Bush D.E.A., LeDoux J.E. (2004). Emotional perseveration: An update on prefrontal-amygdala interactions in fear extinction. Learn. Mem..

[bib36] Spielberger C.D. (1983). Manual for the State-Trait Anxiety Inventory.

[bib37] Tranel D., Bechara A., Denburg N.L. (2002). Asymmetric functional roles of right and left ventromedial prefrontal cortices in social conduct, decision-making, and emotional processing. Cortex.

[bib38] Tsetsenis T., Ma X., Lo Iacono L., Beck S.G., Gross C. (2007). Suppression of conditioning to ambiguous cues by pharmacogenetic inhibition of the dentate gyrus. Nat. Neurosci..

[bib39] Tzourio-Mazoyer N. (2002). Automated anatomical labeling of activations in SPM using a macroscopic anatomical parcellation of the MNI MRI single-subject brain. Neuroimage.

[bib40] Visscher K.M. (2003). Mixed blocked/event-related designs separate transient and sustained activity in fMRI. Neuroimage.

[bib41] Vul E., Harris C., Winkielman P., Pashler H. (2009). Puzzingly High Correlations in fMRI Studies of Emotion, Personality and Social Cognition. Perspect. Psychol. Sci..

[bib42] Wager T.D. (2004). Placebo-induced changes in FMRI in the anticipation and experience of pain. Science.

[bib43] Wager T.D., Davidson M.L., Hughes B.L., Lindquist M.A., Ochsner K.N. (2008). Prefrontal subcortical pathways mediating successful emotion regulation. Neuron.

