# Supplementary material for: Fear-Conditioning Mechanisms Associated with Trait Vulnerability to Anxiety in Humans
Source: Neuron. 2011 Feb 10;69(3):563–71. doi: 10.1016/j.neuron.2010.12.034 (PMC3047792; doi:10.1016/j.neuron.2010.12.034)
Supplement: Document S1. Supplemental Experimental Procedures, Four Figures, and One Table [file mmc1.pdf]

Neuron, Volume *69*

Supplemental Information

Fear-Conditioning Mechanisms Associated with Trait Vulnerability to Anxiety in Humans

Iole Indovina, Trevor W. Robbins, Anwar O. Núñez-Elizalde, Barnaby D. Dunn, and Sonia J. Bishop

Figure S1

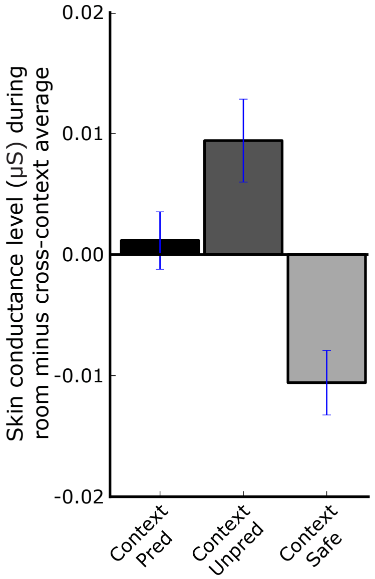

Figure S1. Cross-group skin conductance levels (SCL) during the three contexts, relative to mean across contexts, for the scanning session. Data is natural log transformed. SCL throughout presentation of the unpredictable room was elevated relative to throughout presentation of the safe and predictable rooms, $t(22) = 3.53$, $P < 0.001$, 1-tailed, $t(22) = 1.57$, $P = 0.065$, 1-tailed, respectively. Note. Interference from the scanner gradients led to the CS-specific skin conductance response (SCR) data having lower signal to noise and deviating further from normality than that obtained during the initial acquisition/training session (conducted outside the scanner). Here, non-parametric tests revealed that the SCR to the predictive CS was significantly greater than that to the safe room CS, Wilcoxon $t(22) = 1.67$, $P < 0.05$, 1-tailed, while the SCR to the non-predictive CS did not differ significantly from that to the safe room CS, Wilcoxon $t(22) = 0.91$, $P > 0.1$, 1-tailed.

Figure S2

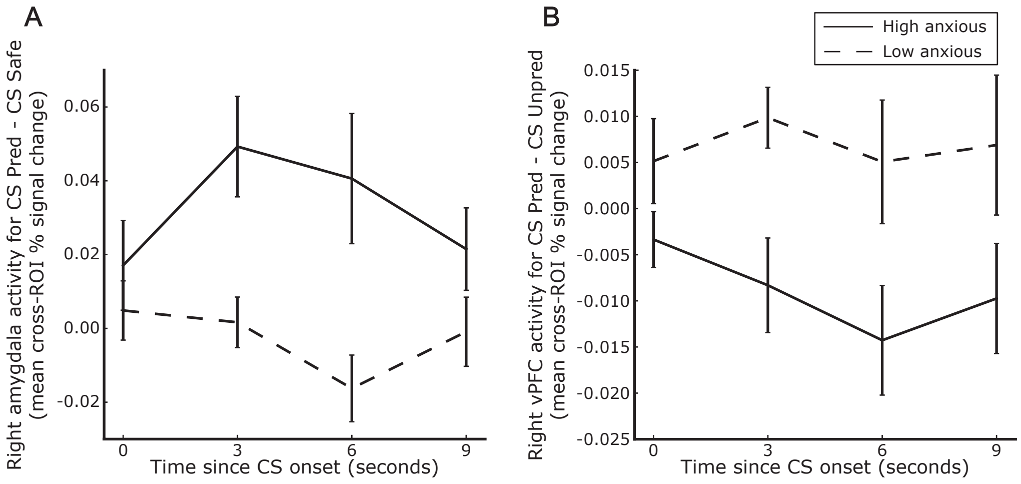

Figure S2. Time courses from the supplementary FIR analysis. A supplementary FIR analysis (see Supplemental Experimental Procedures) was conducted in order to examine the time course of (**A**) the differential amygdala response to the predictive CS versus the safe CS and (**B**) the differential vPFC response to the predictive CS versus the non-predictive CS as a function of trait anxiety. For visualization purposes, in panels A and B, a median split on the STAI trait subscale is used to divide individuals into low and high anxious groups. For the actual FIR analysis, STAI trait scores were entered as a continuous covariate in order to be comparable to the analyses using the canonical HRF reported in the main text. The results of this analysis were as follows.

Amygdala response to predictive CS versus safe CS. At CS onset, there was not a significant difference in amygdala activity to CS pred vs CS safe, across subjects, nor a significant interaction of trait anxiety-entered as a continuous variable—by cue type, *P*s > 0.1. Across the next three time points (3, 6 and 9 seconds post CS onset), there was a significant interaction of trait anxiety by cue type by time bin, $F(1,21) = 5.88$, $P < 0.05$. Separate examination of these time points revealed that there was a significant positive linear effect of trait anxiety upon participants' differential amygdala response to the predictive CS versus safe CS for the second FIR time bin (3 seconds after CS onset), r(21) = 0.44, $P < 0.02$, 1-tailed, and a trend in this direction for the third time bin (6 seconds post CS onset), r(21) = 0.31, $P = 0.07$, 1-tailed, but no significant effect once 9 seconds post CS onset was reached, r(21) = 0.09, $P > 0.3$, 1-tailed. For this fourth time bin, the cross-subject analysis also revealed no differential amygdala response to the predictive CS versus safe CS, $P > 0.2$.

VPFC response to the predictive CS versus the non-predictive CS. The largest effect of STAI trait anxiety upon participants' vPFC response to the predictive CS versus non-predictive CS was also observed in the second FIR time bin. Here, there was a significant negative relationship between trait anxiety, entered as a continuous variable, and phasic recruitment of vPFC to CSpred versus CSunpred, r(21) = -0.54, $P < 0.01$, 1-tailed, replicating the finding from our main analysis using the canonical HRF. In accord with the time course of the effect of trait anxiety upon amygdala activity, there was a trend towards a negative relationship between trait anxiety and phasic recruitment of vPFC to CSpred versus CSunpred in the third FIR time bin, r(21) = -0.33, $P = 0.06$, 1-tailed, but no significant effect of trait anxiety upon the differential vPFC response to CSpred vs CSunpred in time bin 1 (time of CS onset) or time bin 4 (9 seconds post onset), *P*s > 0.1, 1-tailed. The cross-subject analysis also showed no differential vPFC response to CSpred versus CSunpred in these time bins, *P*s > 0.1.

Table S1

Contrast	Correlation with trait anxiety (r)	Significance (P)
Amygdala to CS Pred vs CS Safe	0.44	0.02
Amygdala to CS Pred vs CS Safe (with state anxiety partialled out)	0.37	0.04[a]
Phasic vPFC to CS Pred vs CS Unpred	-0.54	<0.01
Phasic vPFC to CS Pred vs CS Unpred (with state anxiety partialled out)	-0.46	0.01
Sustained vPFC to Context Unpred vs Context Pred	-0.43	0.02
Sustained vPFC to Context Unpred vs Context Pred (with state anxiety partialled out)	-0.37	0.04
**Contrast**	**Correlation with SCR (r)**	**Significance (P)**
Amygdala to CS Pred vs CS Safe	0.55[b]	<0.01
Trait anxiety	0.36[b]	0.04
Trait anxiety controlling for amygdala activity to CS Pred vs CS Safe	0.16[b]	>0.20
Phasic vPFC to CS Pred vs CS Unpred	-0.51[c]	0.01
Sustained vPFC to Context Unpred vs Context safe	-0.38[c]	0.04[a]

Table S1. Results from the supplementary FIR analysis. The relationship of trait anxiety to both phasic and sustained indices of regional neural activity replicates the findings from the analyses using the canonical HRF reported in the main text. Specifically, using the 2nd time bin, results for the cue-related contrasts from the primary analyses are replicated. The effects of trait anxiety upon sustained vPFC recruitment and its relationship to skin conductance levels are also replicated. CS events were modelled by FIR functions, UCS and context regressors by step functions convolved with the canonical HRF. Additional analyses with UCS events also modelled using FIR functions were conducted, all results reported here remained significant at $P < 0.05$ except for those indicated by (a) which trended towards significance at $0.05 < P < 0.10$. (b) Skin conductance from early acquisition session. (c) Skin conductance from late acquisition/expression session.

Figure S3

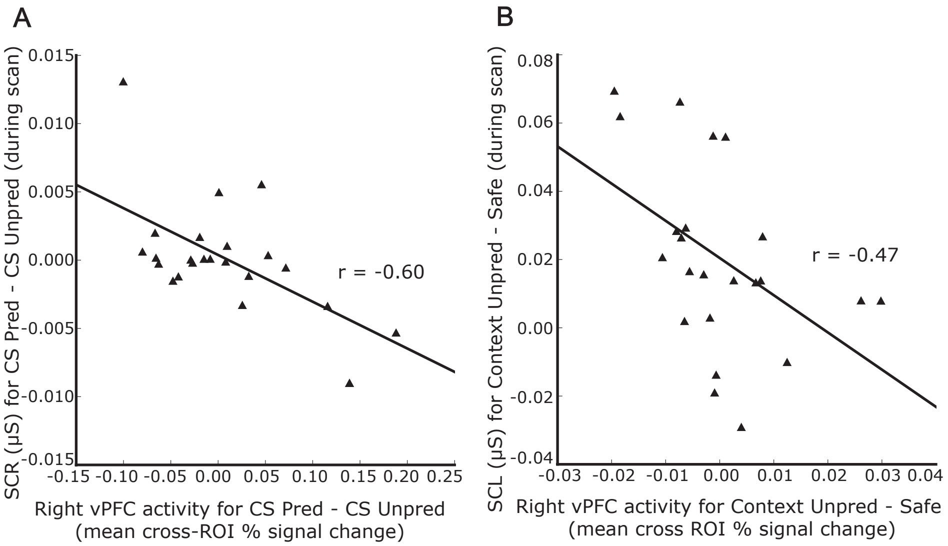

Figure S3. Elevated phasic and sustained vPFC activity during conditioned fear expression were associated with reduced cued and contextual fear, respectively. (**A**) Phasic vPFC activity to the predictive versus non-predictive CS was inversely correlated with SCR to the predictive versus non-predictive CS. (**B**) Increased vPFC activity during the unpredictable (versus safe) room was similarly associated with reduced concurrent skin conductance levels across this room (relative to the 'safe' room). Note. Cross-ROI % signal change is derived from the canonical HRF analyses as reported in the main text.

Figure S4

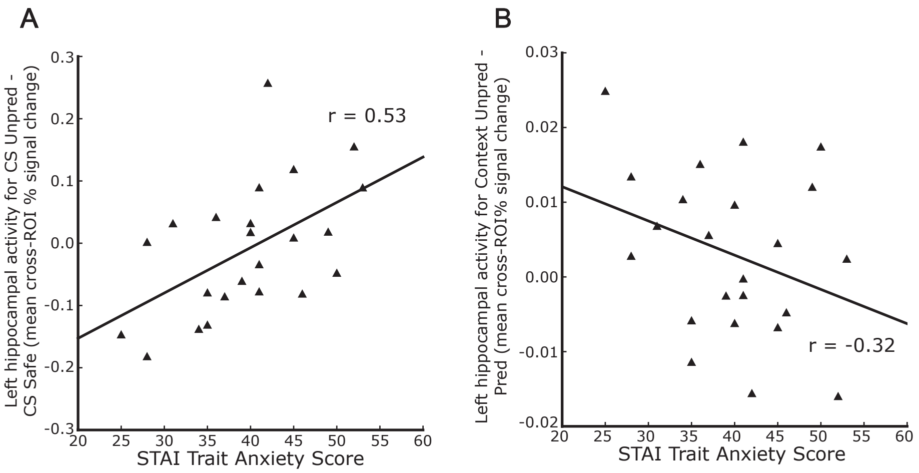

Figure S4 Trait anxiety modulated phasic and sustained hippocampal activity during cued and contextual fear conditioning. (**A**). Across participants, increased hippocampal activity was observed to the predictive CS relative to the safe room CS, t(22) = 3.10, p=.005, 2-tailed. Elevated anxiety was associated with phasic increase in hippocampal activity also being observed to the unpredictable CS relative to the safe room CS (activity plotted is that averaged across all voxels within the left MNI AAL hippocampal ROI, a significant correlation was also observed in the right ROI, r(21) = 0.42, P < 0.05, 2-tailed.) (**B**) There was also a trend towards elevated trait anxiety being associated with reduced sustained hippocampal activity across the unpredictable room relative to the predictable room. Note. Cross-ROI % signal change is derived from the canonical HRF analyses as reported in the main text.

Supplemental Experimental Procedures

Supplementary FIR analyses.

In order to examine the time course of the phasic amygdala and vPFC responses we conducted supplementary analyses where we substituted finite impulse response (FIR) functions to model the cue effects (this gives *n* separate regressors corresponding to *n* time points following CS onset for each CS type). To preserve the benefit of jittering between CS and UCS onsets, linear interpolation was used to enable modeling of event occurrence with a temporal resolution of greater than 1 TR (3 seconds). This was achieved by using the temporal proximity of the two neighboring scans (defined as the beginning of volume acquisition) of a given event to weight the values corresponding to those scans for each FIR time bin regressor and normalizing the sum of those weights to one. Following Visscher et al. (2003), block regressors were modeled with a single boxcar function (a step function of length defined by the room duration) convolved with the canonical HRF (this is similar to their convolution with a single gamma function, but also models the BOLD undershoot). Alternate analyses were conducted modeling the UCS (scream) either as a step function convolved with the canonical HRF or using FIR functions for this event type as well. The two analyses gave similar results (**Table S1**). We used a FIR model with 4 time bins given that addition of further time bins did not lead to a significant change in variance explained by the model – mean ΔR^2 across subjects for our ROIs < 0.01%, *P*s > 0.3. (It is of note that a recently published methodological report on FIR analysis similarly found a FIR model which focused on 0-9 seconds after event onset to be optimal to avoid overfitting, Kay et al., 2008).

Modeling of the UCS.

In both our primary analyses and the supplementary FIR analysis, UCS (scream) occurrences were modeled by a single regressor. This enables activity to the CS in the predictable and unpredictable rooms to be more easily dissociated from that to the UCS. This approach relies upon the response to the UCS being similar across the predictable and unpredictable rooms. To explore this further, we examined both the skin conductance response and the amygdala response to the UCS in the predictable room relative to that in the unpredictable room. Neither of these measures differed significantly by room type – SCR: t(21) = 1.22 , *P* > 0.2, left amygdala: t(21) = 1.24, *P* > 0.2, right amygdala: t(21) = 0.49, *P* > 0.5, all tests 2-tailed. Further, with the screams modeled separately for each room, while collinearity of regressors increased, the relationship between amygdala activity to the predictive CS vs safe CS and trait anxiety remained significant, r(21) = 0.41, *P* < 0.05.

Supplemental Reference

Kay, K.N., David, S.V., Prenger, R.J., Hansen, K.A. and Gallant, J.L. (2008) Modeling low-frequency fluctuation and hemodynamic response timecourse in event-related fMRI. Hum Brain Mapp. *29*, 142-156.
